# Oxygen-containing functional group-facilitated CO_2_ capture by carbide-derived carbons

**DOI:** 10.1186/1556-276X-9-189

**Published:** 2014-04-23

**Authors:** Wei Xing, Chao Liu, Ziyan Zhou, Jin Zhou, Guiqiang Wang, Shuping Zhuo, Qingzhong Xue, Linhua Song, Zifeng Yan

**Affiliations:** 1 State Key Laboratory of Heavy Oil Processing, School of Science, China University of Petroleum, Qingdao 266580, People's Republic of China; 2 School of Chemical Engineering, Shandong University of Technology, Zibo 255049, People's Republic of China

**Keywords:** Carbide-derived carbons, CO_2_ adsorption, Oxidation

## Abstract

A series of carbide-derived carbons (CDCs) with different surface oxygen contents were prepared from TiC powder by chlorination and followed by HNO_3_ oxidation. The CDCs were characterized systematically by a variety of means such as Fourier transform infrared spectroscopy, X-ray photoelectron spectroscopy, ultimate analysis, energy dispersive spectroscopy, N_2_ adsorption, and transmission electron microscopy. CO_2_ adsorption measurements showed that the oxidation process led to an increase in CO_2_ adsorption capacity of the porous carbons. Structural characterizations indicated that the adsorbability of the CDCs is not directly associated with its microporosity and specific surface area. As evidenced by elemental analysis, X-ray photoelectron spectroscopy, and energy dispersive spectroscopy, the adsorbability of the CDCs has a linear correlation with their surface oxygen content. The adsorption mechanism was studied using quantum chemical calculation. It is found that the introduction of O atoms into the carbon surface facilitates the hydrogen bonding interactions between the carbon surface and CO_2_ molecules. This new finding demonstrated that not only the basic N-containing groups but also the acidic O-containing groups can enhance the CO_2_ adsorbability of porous carbon, thus providing a new approach to design porous materials with superior CO_2_ adsorption capacity.

## Background

Observational evidence proved that global warming has already caused a series of severe environmental problems such as sea level rise, glacier melt, heat waves, wildfires, etc. [1,2]. These disasters have already greatly damaged the balance of nature. It is widely believed that the global warming in recent years is mainly ascribed to the excessive emission of greenhouse gases, in which CO_2_ is the most important constituent. According to the Fourth Assessment Report which was published by Intergovernmental Panel on Climate Change (IPCC) in 2007, the annual emissions of CO_2_ have grown from 21 to 38 gigatonnes (Gt) and the rate of growth of CO_2_ emissions was much higher during 1995 to 2004 (0.92 Gt per year) than that of 1970 to 1994 (0.43 Gt per year) [3]. So, it is urgent to develop CO_2_ capture and storage (CCS) technologies [4].

In an early stage, people used to trap CO_2_ in some geological structures such as depleted oil and gas reservoirs, deep saline aquifers, unminable coal beds, etc. [5-7]. However, CO_2_ geological storage usually requires large-scale equipment which calls for great costs. On the other hand, there is an obstacle to reuse the CO_2_, which has been trapped in these geological structures, as an industrial raw material due to its low purity grade. So, it is necessary to develop a more feasible CCS technology.

The application of porous materials in the capture and storage of CO_2_ has a big potential and wide prospect. There are many kinds of porous materials that can be used as CO_2_ adsorbents, such as molecular sieves, porous silica, metal organic frameworks (MOFs), and porous carbons [8-18] due to their attractive properties such as high specific surface area and highly developed pore structure. Among these porous materials, porous carbons are especially attractive because they are inexpensive, easy to regenerate, and not sensitive to moisture which may compete with CO_2_ when adsorption happens [19-21]. However, it is hard for pristine porous carbon materials without any modification to reach high CO_2_ uptake values [22]. As a result, researchers modified the surface of porous carbon with nitrogen-containing functional groups [23], which enhanced the CO_2_ adsorption capacity of these porous carbon materials. For example, Chandra et al. synthesized a kind of N-doped carbon by chemical activation of polypyrrole functionalized graphene sheets. This kind of carbon material showed a CO_2_ uptake of 4.3 mmol g^−1^ with high selectivity at 298 K under 1 atm [24]. Zhou et al. prepared a series of N-doped microporous carbons using zeolite NaY as a hard template and furfuryl alcohol/acetonitrile as carbon precursors. The CO_2_ adsorption capacity of as-prepared N-doped carbons was much higher than that of the template carbons without N-doping [25]. Nandi et al. prepared a series of highly porous N-doped activated carbon monoliths by physical activation. The monoliths exhibit an excellent CO_2_ uptake of up to 5.14 mmol g^−1^ at ambient temperature and 11.51 mmol g^−1^ at 273 K under atmospheric pressure [26]. Wu et al. synthesized a series of nitrogen-enriched ordered mesoporous carbons via soft-template method. The CO_2_ adsorption capacity of nitrogen-enriched carbon is higher than that of pristine material due to the presence of nitrogen-containing functionalities [27]. Sevilla et al. prepared a series of N-doped porous carbons using KOH as activation agent and polypyrrole as carbon precursor. The excellent CO_2_ uptakes of these carbons were ascribed to the abundant micropores with the pore size around 1 nm and the presence of basic N-containing groups [19]. Hao et al. synthesized a kind of nitrogen-containing carbon monolith through a self-assembled polymerization of resol and benzoxazine followed by carbonization. The high CO_2_ adsorption capacity was attributed to the N-containing groups of the resulting carbons [21]. The above-mentioned works all proved that the presence of nitrogen-containing functional groups can enhance the CO_2_ adsorption capacities of porous carbons, and all these authors simply attribute this adsorption-enhancing effect to the acid-base interactions between acidic CO_2_ molecules and basic N-containing groups without providing any experimental evidence. However, for these N-doped porous carbons that are prepared at high temperatures, the N atoms reside in the carbon skeleton and are stable at high temperatures. The basicity of these N-containing functional groups is very much weaker than that of organic amines and is rarely studied in the literatures. To the best of our knowledge, there is no direct experimental evidence to prove that this acid-base interaction does exist between CO_2_ molecules and the N-containing groups of the N-doped carbon. Our previous research has proved that this CO_2_ adsorption-enhancing effect for N-doped carbon is due to the hydrogen bonding interactions between CO_2_ molecules and H atoms on the carbon surface. This hydrogen bonding interactions are facilitated efficiently by N-doping, which challenges the acid-base interacting mechanism generally accepted in this field [28].

In this paper, the influence of oxygen-containing groups of the porous carbon on CO_2_ capture property is studied for the first time. It is found that the presence of oxygen-containing functional groups can enhance the CO_2_ adsorption capacity of porous carbons. As evidenced by both quantum chemical calculations and a variety of characterization means, this adsorption-enhancing effect is attributed to the hydrogen bond interactions between hydrogen atoms on the carbon surface and CO_2_ molecules, which is greatly enhanced by the presence of O atoms on the carbon surface. As we know, most oxygen-containing functional groups such as phenolic hydroxyl groups, carboxyl groups, lactone groups, and aldehyde groups show acid tendency [29]. According to the acid-base interacting mechanism currently accepted in this field, the presence of such acidic groups would show a negative effect on CO_2_ adsorption. Therefore, our work challenges the acid-base interacting mechanism currently accepted in this field. Our new finding also provides a new approach to design porous materials with superior CO_2_ adsorption capacity.

## Methods

### Material preparation

The carbide-derived carbons (CDCs) were prepared by chlorinating TiC according to the literatures [30,31]. In the preparation, the TiC powder was placed in a quartz boat and then loaded into a quartz tube furnace. First, the quartz tube with a quartz boat inside was purged with nitrogen to thoroughly dispel oxygen. Then, the temperature of the furnace was raised to 700°C by 5°C min^−1^ under nitrogen flow (40 mL min^−1^). Afterwards, the nitrogen flow was shifted to chlorine flow (15 mL min^−1^) for 3 h. The resulting powder was annealed under hydrogen at 600°C for 2 h to remove residual chlorine and chlorine-containing compounds.

To investigate the influence of oxygen content on CO_2_ adsorption capacity, the as-prepared CDC was placed in a flask followed by the addition of 25 mL concentrated nitric acid for oxidation. After stirring under different temperatures for 3.5 h, the obtained carbon powder was washed thoroughly with deionized water. The dried sample was named as CDC-*x*, where *x* represents the oxidation temperature. The reduced carbon samples were obtained by heating CDC-*x* in H_2_ atmosphere at 800°C for 3 h and were denoted as CDC-*x*-HR.

### Material characterization

The pore structure parameters and CO_2_ adsorption capacities of the carbon samples were analyzed with a surface area and porosity analyzer (ASAP 2020, Micromeritics Corp., Norcross, GA, USA). Nitrogen sorption isotherms and CO_2_ adsorption isotherms were determined at 77 and 298 K, respectively. The carbon samples were strictly degassed under vacuum (0.2 Pa) at 350°C overnight before sorption measurements. N_2_ and CO_2_ gases with super high purity (99.999%) were used for the sorption measurements. The specific surface area and micropore volumes of the carbons were measured by Brunauer-Emmett-Teller (BET) method and *t*-plot method, respectively. The single-point total pore volume was measured at *p*/*p*_0_ = 0.995 and the average pore size was equal to 4*V*_total_/*S*_BET_. Microscopic morphologies of the carbons were observed using a transmission electron microscope (TEM, Hitachi H800, Chiyoda, Tokyo, Japan). The chemical compositions of the carbons were determined using both a Vario EI IIIb element analyzer and an energy dispersive spectrometer (EDS; INCA Energy, Oxford, Buckinghamshire, UK). The surface chemical property of the carbons was analyzed by a X-ray photoelectron spectroscope (XPS; PHI-5000 Versaprobe, Chanhassen, MN, USA) using a monochromated Al Kα excitation source. The binding energies were calibrated with respect to C1s (284.6 eV). Fourier transform infrared spectroscopy (FT-IR) analyses were carried out on a Nicolet 5800 infrared spectrometer (Madison, WI, USA) with an accuracy of 0.09 cm^−1^. The carbons were first mixed with KBr at a mass ratio of 1/100 and then ground in an agate mortar for pressing KBr pellets.

## Results and discussion

### Surface properties and pore structure of carbon samples

FT-IR was used to identify oxygen-containing functional groups of the CDC samples. Compared with the pristine CDC sample before oxidation, the FT-IR spectrum of CDC-50 (Additional file 1: Figure S1) shows some new characteristic bands that were introduced by HNO_3_ oxidation. The band at 3,200 to 3,600 cm^−1^ was attributed to hydroxyl groups. The band at around 1,710 cm^−1^ was attributed to -C = O stretching vibration. The peaks between 1,000 to 1,300 cm^−1^ can be assigned to -C-O stretching and -OH bending modes of alcoholic, phenolic, and carboxylic groups. All this new emerging bands indicate that HNO_3_ oxidation introduced a large number of oxygen-containing functional groups, such as hydroxyl, carbonyl, and carboxyl groups, to the CDC [32-34].

Moreover, elemental analysis (EA), EDS, and XPS were employed to intensively investigate the oxygen content of the carbons. As is shown in Table 1, all these three characterization means undoubtedly demonstrate that HNO_3_ oxidation did introduce a large quantity of oxygen atoms to the carbon; HNO_3_ oxidation at higher temperature would introduce more oxygen atoms to the carbon; the subsequent H_2_ reduction can effectively remove oxygen atoms from the oxidized carbons. For instance, as for EA data, the oxygen content of the carbons increased from 17.6 to 36.7 wt% and 41.5 wt% after oxidizing pristine CDC by HNO_3_ at 50°C and 80°C, respectively. The subsequent H_2_ reduction decreased the oxygen contents to 11.2 and 20.5 wt% for CDC-50 and CDC-80, respectively.

**Table 1:** **Specific surface areas**, **pore structure parameters, and oxygen contents of CDCs**

**Sample**	***S***_**BET**_^**a**^	***V***_**micro**_^**b**^	***V***_**total**_^**c**^	**Pore size**^**d**^	**O content**		
	**(m**^**2**^ **g**^−**1**^**)**	**(cm**^**3**^ **g**^−**1**^**)**	**(cm**^**3**^ **g**^**−1**^**)**	**(nm)**	**EA (wt%)**	**XPS (wt%)**	**EDS (wt%)**
Pristine CDC	1,216	0.59	0.65	2.13	17.6	8.7	6.8
CDC-50	907	0.43	0.47	2.06	36.7	14.6	20.3
CDC-50-HR	1,115	0.51	0.58	2.08	11.2	10.2	10.3
CDC-80	449	0.22	0.24	2.15	41.5	15.7	29.8
CDC-80-HR	497	0.22	0.27	2.21	20.5	14.2	16.0

^a^BET specific surface area. ^b^Micropore volumes calculated by the *t*-plot method. ^c^Single-point total pore volume measured at *p*/*p*_0_ = 0.995. ^d^Pore size = 4*V*_total_/*S*_BET_.

Nitrogen physisorption measurements were performed at 77 K to characterize the surface areas and pore structures of CDCs. The N_2_ adsorption isotherms of all the carbons (Additional file 1: Figure S2) exhibit type I isotherms, and no hysteresis loop can be observed for these samples, indicating the microporous nature of these carbons and the absence of mesopores. The detailed specific surface area and pore structure parameters of these carbons are listed in Table 1. The specific surface area and micropore volume decrease from 1,216 m^2^/g and 0.59 cm^3^/g to 907 m^2^/g and 0.43 cm^3^/g, respectively, after oxidizing the pristine CDC by HNO_3_ at 50°C, which is due to the introduction of oxygen-containing groups to the pore surface of the carbon. After H_2_ reduction, the specific surface area and micropore volume increase back to 1,115 m^2^/g and 0.51 cm^3^/g, indicating that the oxygen-containing groups are effectively removed from the pore surface by H_2_ reduction. This result coincides with the elemental analyses data. It is also suggested that the oxidation of the pristine CDC by HNO_3_ at 50°C did not obviously damage the pore structure of the carbon and that the decrement in the specific surface area and micropore volume due to the oxidation can be mostly recovered by H_2_ reduction. By contrast, oxidizing the pristine CDC by HNO_3_ at 80°C results in the dramatic decrease of the specific surface area and micropore volume. Although the subsequent H_2_ reduction can effectively remove oxygen-containing groups from CDC-80, the surface area and micropore volume cannot be recovered, indicating that HNO_3_ oxidation at 80°C severely damaged the micropore structure of the carbon.

**Figure 1:**
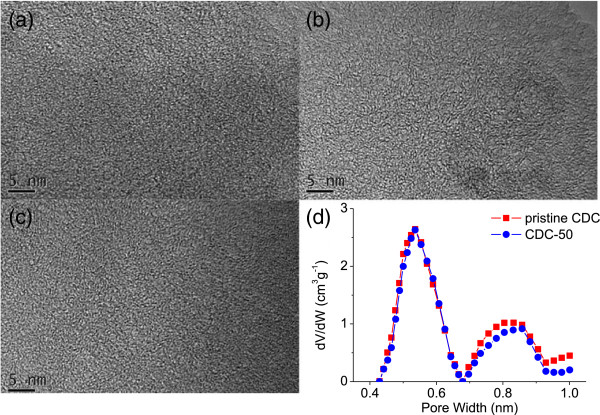
TEM images of CDCs: (a) CDC, (b) CDC-50, and (c) CDC-80, and (d) micropore size distribution of CDCs.

In order to further clarify the pore structure evolution caused by HNO_3_ oxidation, TEM observations were also conducted to get the microscopic morphology of the CDC. The pristine CDC (Figure 1a) shows amorphous structure and abundant micropores that are formed by the stacking of curved graphene layers. The samples CDC-50 and CDC-80 (Figure 1b,c) show similar microscopic morphology to the pristine CDC, suggesting the microporous nature of all the three samples. These results coincide with the pore size data shown in Table 1.

### CO_2_ capture performances of the CDCs

**Figure 2:**
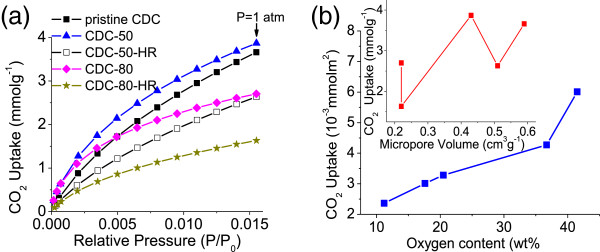
**CO**_**2 **_**adsorption isotherms for the CDCs (a) and a plot of CO**_**2 **_**uptake vs. oxygen content (b).** The inset is a plot of CO_2_ uptake vs. micropore volume.

According to classical gas adsorption theories, gas adsorption on porous carbons usually relies on the highly developed microporous structure and large specific surface area. Recent studies also demonstrated that micropores (<1 nm) are beneficial to CO_2_ adsorption for porous materials [18,35-38]. In this work, CDC-50 shows lower specific area and micropore volume (Table 1 and Figure 1d) than the pristine CDC and CDC-50-HR. However, as shown in Figure 2a, CDC-50 (3.87 mmol g^−1^ under 1 atm) possesses an apparently higher CO_2_ uptake than the pristine CDC (3.66 mmol g^−1^ under 1 atm) and CDC-50-HR (2.63 mmol g^−1^ under 1 atm). Likewise, CDC-80 has a lower specific surface area and the same micropore volume than/as its reduced product CDC-80-HR. However, the former (2.71 mmol g^−1^ under 1 atm) possesses an obviously higher CO_2_ uptake than the latter (1.63 mmol g^−1^ under 1 atm). As for CDCs, their CO_2_ uptakes do not have a linear correlation with their micropore volume, as is shown in Figure 2b inset. So, the CO_2_ adsorption results for the CDCs cannot be explained by classical adsorption theories. Nevertheless, it is very instructive to find that the CO_2_ uptakes per unit surface area of the carbons are positively related to the oxygen content of the carbons (Figure 2b), indicating that the CO_2_ adsorption capacity of the carbons was greatly facilitated by the introduction of oxygen-containing groups to the carbon. This result agrees well with the work of Liu [5].

**Figure 3:**
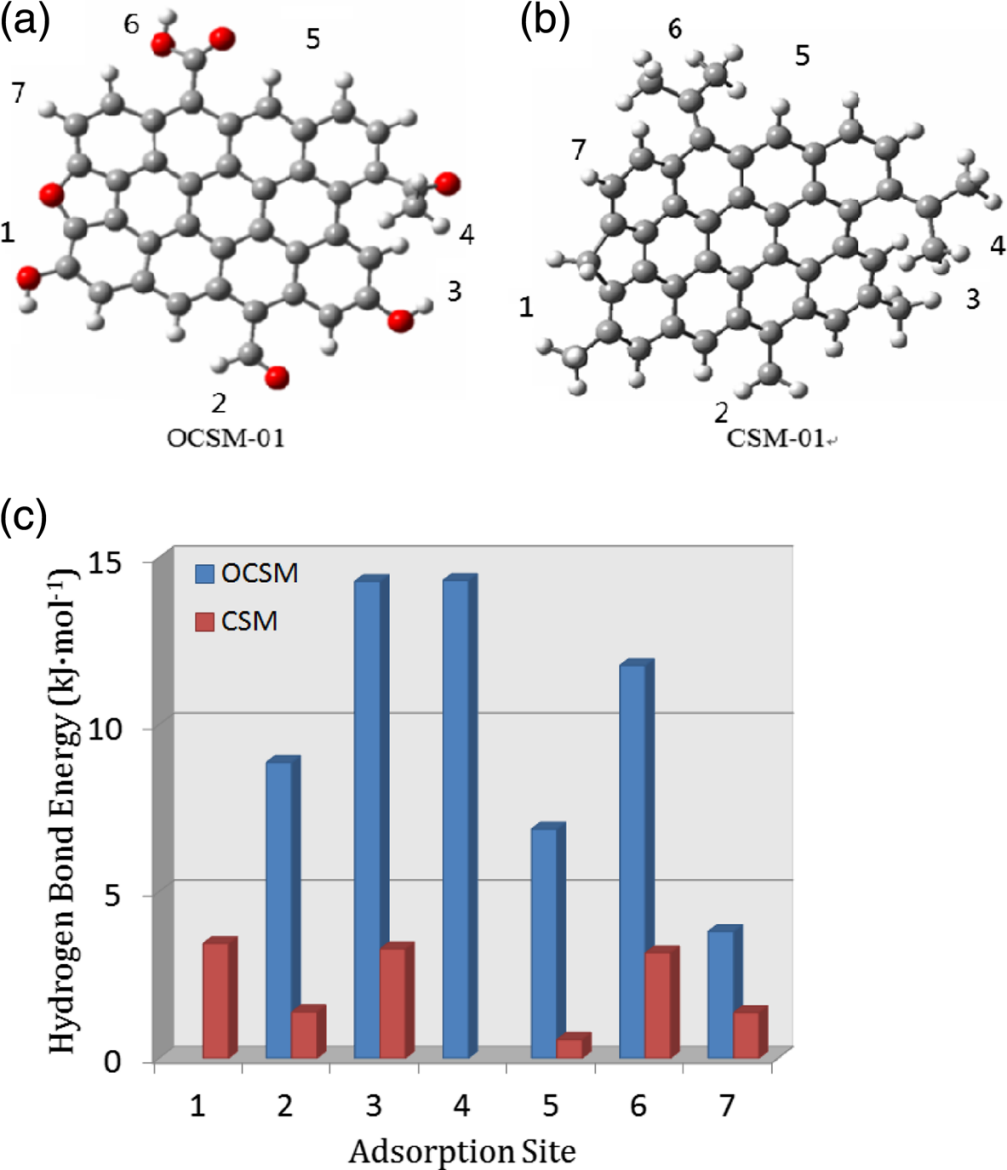
**Theoretical carbon models and hydrogen bond energies.** Theoretical models for **(a)** oxygen**-**containing carbon surface and **(b)** pure carbon surface (red ball: oxygen atom; grey ball: carbon atom; small grey ball: hydrogen atom). **(c)** Hydrogen bond energies at different adsorption sites.

In order to reveal the effect of oxygen-containing groups on CO_2_ adsorption for the carbons, a theoretical carbon surface model (OCSM) containing six different typical O-containing functional groups was developed in light of Niwa's model [39]. A pure carbon model without oxygen atoms (CSM) was also devised for comparison, as is shown in Figure 3. Density functional theory B3LYP was employed to study the interactions between these models and CO_2_, and all the configurations were optimized with the 6-31 + G* basis set for all atoms using the Gaussian-03 suite package [40].

The optimized results at the 6-31 + G* level are that there are six OCSM-CO_2_ complexes and six CSM-CO_2_ complexes. Furthermore, the calculated results demonstrate that the frequency values of all complexes are positive, showing that they are in stable configurations. Additional file 1: Figure S3 illustrates the geometric configurations for all the complexes, and Additional file 1: Table S1 tabulates the total energies for all the complexes. In these complexes, hydrogen bonds between CO_2_ and OCSM/CSM are formed due to the high electronegativity of the oxygen atom in the CO_2_ molecule. This type of weak hydrogen bond has been widely studied in recent years. The experimental and theoretical studies have demonstrated its existence although the interaction of C-H · · · O is weaker than that of typical hydrogen bonds such as O-H · · · O and N-H · · · O [41-43].

Computational results indicated that the binding energies for such hydrogen bonds are different at various positions. It is apparent that the larger the bonding energy Δ*E* (kJ mol^−1^), the stronger the adsorption affinity. The average binding energy of six OCSM-CO_2_ complexes is 9.98 kJ mol^−1^, and that of CSM-CO_2_ complexes is 2.20 kJ mol^−1^, suggesting that the hydrogen bonds in the OCSM-CO_2_ complexes are much stronger than those in CSM-CO_2_ complexes. This binding energy difference (7.78 kJ mol^−1^) between OCSM-CO_2_ and CSM-CO_2_ complexes roughly agrees with the difference of CO_2_ adsorption heat between the pristine CDC and CDC-50 (as shown in Additional file 1: Figure S4), which somewhat reflects the effect of oxygen introduction on CO_2_ adsorption heat for the CDCs.

**Figure 4:**
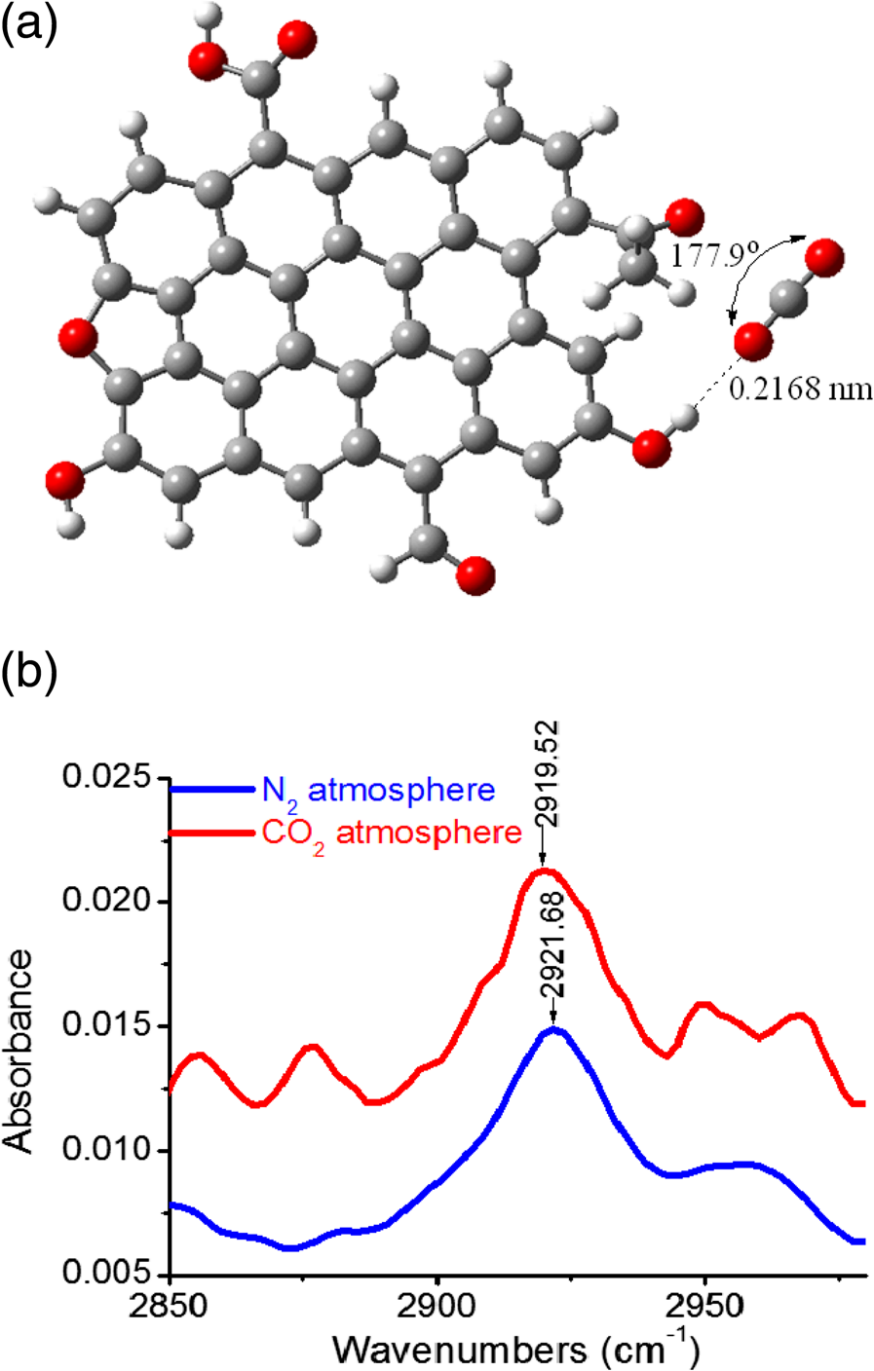
**Hydrogen bonding interaction and FT**-**IR spectra. (a)** The interaction between the theoretical model of CDC surface and CO_2_ molecule and **(b)** FT**-**IR spectra of CDC**-**50 measured under different atmospheres.

In order to prove the existence of the hydrogen bonding interactions between the carbon and CO_2_ molecules, FT-IR spectra (Figure 4) were recorded for CDC-50 under both N_2_ and CO_2_ atmospheres using a Nicolet 5700 infrared spectrometer with an accuracy of 0.1 cm^−1^. Under N_2_ atmosphere, the peak at 2,921.68 cm^−1^ was attributed to the C-H anti-symmetric stretching vibration. When the atmosphere was shifted to CO_2_, this peak was broadened and redshifted to low wavenumber, 2,919.52 cm^−1^. The already published papers proved that hydrogen bonding interactions can weaken the C-H bonding energy, which lead to the redshift of corresponding peak on the FT-IR spectra [44,45]. This phenomenon confirms that the hydrogen bonding interactions between CDC-50 and CO_2_ molecules do exist. Unfortunately, due to the interference caused by adsorbed water moisture on the carbon samples in FT-IR measurements, the effects of hydrogen bonding on O-H and C-O bonds cannot be observed. Besides, elemental analyses show that HNO_3_ oxidation can increase the H content from 13 to 33 mmol g^−1^ for the pristine CDC and CDC-50, respectively, which enables more hydrogen bonding interactions between CDC-50 and CO_2_ molecules. This also explains why the oxidized CDC samples possess higher CO_2_ uptakes.

## Conclusions

We intensively investigated the effect of introducing oxygen-containing functional groups to the carbon surface on the CO_2_ uptake of CDCs. Structural characterizations and CO_2_ adsorption on the CDCs indicate that CO_2_ uptake is independent of the specific surface area and micropore volume of the CDCs but closely related to the oxygen content of the carbons. Quantum chemical calculations and FT-IR measurements reveal that the introduction of oxygen atoms into a carbon surface facilitates the hydrogen bonding interactions between the carbon surface and CO_2_ molecules, which accounts for the enhanced CO_2_ uptake on the oxidized CDCs. Because most oxygen-containing functional groups show acidic tendency, this new finding challenges the ‘acid-base interacting mechanism’ generally accepted in this field. This new finding also provides a new approach to design porous carbon with superior CO_2_ adsorption capacity.

## Competing interests

The authors declare that they have no competing interests.

## Authors’ contributions

WX and CL performed the experiments and drafted the manuscript together. ZZ performed the CO_2_ adsorption simulation. JZ and GW checked the figures and gave the final approval of the version to be published. SZ, QX, and LS performed the partial experiments. ZY guided the idea and revised and finalized the manuscript. All authors read and approved the final manuscript.

## Supplementary Material

Additional file 1 **Supporting information. Table S1.** the total energies for OCSM-CO_2_ and CSM-CO_2_ complexes. **Table S2.** chemical composition of the CDCs determined by elemental analysis. **Figure S1.** FT-IR spectra of pristine CDC and CDC-50. **Figure S2.** nitrogen adsorption isotherms of the CDCs. **Figure S3.** geometric configurations and total energies for OCSM, CSM, OCSM-CO_2_ complexes and CSM-CO_2_ complexes. **Figure S4.** isosteric heats of CO_2_ adsorption on the carbons at different CO_2_ uptakes. Click here for file
