# Supplementary material for: Oxygen-containing functional group-facilitated CO2 capture by carbide-derived carbons
Source: Nanoscale Res Lett. 2014 Apr 23;9(1):189. doi: 10.1186/1556-276X-9-189 (PMC4021581; doi:10.1186/1556-276X-9-189)
Supplement: Additional file 1 — Supporting information. Table S1. the total energies for OCSM-CO2 and CSM-CO2 complexes. Table S2. chemical composition of the CDCs determined by elemental analysis. Figure S1. FT-IR spectra of pristine CDC and CDC-50. Figure S2. nitrogen adsorption isotherms of the CDCs. Figure S3. geometric configurations and total energies for OCSM, CSM, OCSM-CO2 complexes and CSM-CO2 complexes. Figure S4. isosteric heats of CO2 adsorption on the carbons at different CO2 uptakes. [file 1556-276X-9-189-S1.doc]

**Supporting Information**

**Oxygen-containing functional group facilitated co_2_ capture by carbide derived carbons**

By Wei Xing, * ^a^ Chao Liu, ^a^ Ziyan Zhou,^b^ Jin Zhou, ^b^ Guiqiang Wang,^b^ Shuping Zhuo,* ^b^ Qingzhong Xue, ^a^ Linhua Song,^a^ Zifeng Yan* ^a^

*^a^* School of Science

State Key Laboratory of Heavy Oil Processing

China University of Petroleum

Qingdao 266580 (P. R. China)

Correspondance: [xingwei@upc.edu.cn](mailto:xingwei@upc.edu.cn); [zfyancat@upc.edu.cn](mailto:zfyancat@upc.edu.cn)

*^b^* School of Chemical Engineering

Shandong University of Technology

Zibo 255049 (P. R. China)

E-mails:

**Table S1** The total energies for OCSM-CO_2_ and CSM-CO_2_ complexes

**Table S2** Chemical composition of the CDCs determined by elemental analysis

**Figure S1** FT-IR spectra of pristine CDC and CDC-50

**Figure S2** Nitrogen adsorption isotherms of the CDCs

**Figure S3** Geometric configurations and total energies for OCSM, CSM, OCSM-CO_2_ complexes and CSM-CO_2_ complexes

**Figure S4** Isosteric heats of CO_2_ adsorption on the carbons at different CO_2_ uptakes

Table S1 The total energies for OCSM-CO_2_ and CSM-CO_2_ complexes

|  | *E*_zero_(a.u.) | *E*_总_(a.u.) | △*E*  (kJ/mol) |  | *E*_zero_(a.u.) | *E*_总_(a.u.) | △*E*  (kJ/mol) |
| --- | --- | --- | --- | --- | --- | --- | --- |
| CO_2_ | 0.0115650 | -188.5903926 |  |  |  |  |  |
| OCSM-01 | 0.4486430 | -2060.5975874 |  | CSM-01 | 0.6297060 | -1772.2793706 |  |
| OCSM-03 | 0.4610460 | -2249.1921941 | 8.86 | CSM-02 | 0.6414120 | -1960.8712149 | 3.44 |
| OCSM-04 | 0.4613150 | -2249.1945260 | 14.28 | CSM-03 | 0.6412600 | -1960.8702815 | 1.39 |
| OCSM-05 | 0.4613240 | -2249.1945445 | 14.31 | CSM-04 | 0.6417180 | -1960.8714586 | 3.28 |
| OCSM-06 | 0.4609250 | -2249.1913127 | 6.87 | CSM-06 | 0.6416280 | -1960.8703352 | 0.56 |
| OCSM-07 | 0.4613700 | -2249.1936257 | 11.77 | CSM-07 | 0.6411700 | -1960.8708660 | 3.16 |
| OCSM-08 | 0.4605620 | -2249.1897819 | 3.80 | CSM-08 | 0.6414250 | -1960.8704379 | 1.37 |
| 平均值 |  |  | 9.98 | 平均值 |  |  | 2.20 |

△*E*= [E(M)+E(CO_2_)]-E(M-CO_2_)

Table S2 Chemical composition of the CDCs determined by elemental analysis

| Sample | N(mmol/g) | C(mmol/g) | H(mmol/g) | O(mmol/g) |
| --- | --- | --- | --- | --- |
| Pristine CDC | 1.0 | 52.8 | 13.0 | 11.0 |
| CDC-50 | 1.1 | 46.0 | 33.0 | 22.9 |
| CDC-50-HR | 1.2 | 69.9 | 12.0 | 7.0 |
| CDC-80 | 1.0 | 43.5 | 28.0 | 25.9 |
| CDC-80-HR | 0.7 | 69.8 | 15.0 | 12.8 |

Figure S1 FT-IR spectra of pristine CDC and CDC-50

Figure S2 Nitrogen adsorption isotherms of the CDCs


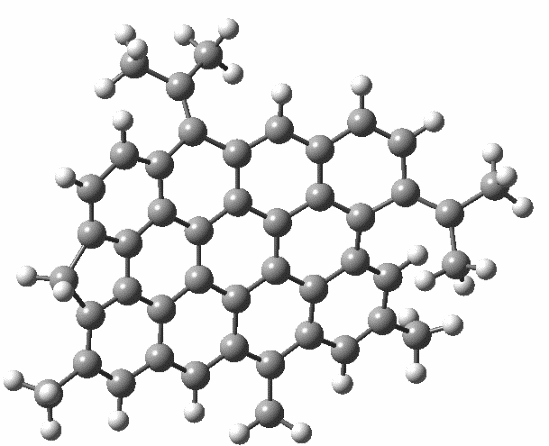


OCSM-01 CSM-01

CSM-02

OCSM-03 CSM-03

OCSM-04 CSM-04

OCSM-05

OCSM-06 CSM-06

OCSM-07 CSM-07

OCSM-08 CSM-08

Figure S3 Geometric configurations and total energies for OCSM, CSM, OCSM-CO_2_ complexes and CSM-CO_2_ complexes

Figure S4 Isosteric heats of CO_2_ adsorption on the carbons at different CO_2_ uptakes
